# Mobile phone sleep self-management applications for early start shift workers: A scoping review of the literature

**DOI:** 10.3389/fpubh.2022.936736

**Published:** 2022-08-10

**Authors:** Ben Bullock, Caitlin Learmonth, Hilary Davis, Abdullah Al Mahmud

**Affiliations:** ^1^Centre for Mental Health, Swinburne University of Technology, Hawthorn, VIC, Australia; ^2^Centre for Social Impact, Swinburne University of Technology, Hawthorn, VIC, Australia; ^3^Centre for Design Innovation, Swinburne University of Technology, Hawthorn, VIC, Australia

**Keywords:** shift work, sleep self-management, sleep technology, mobile app, well-being

## Abstract

Poor sleep has significant impacts on both mental and physical well-being. This is especially the case for shift workers who rely on good sleep practices to manage the disruption caused by their working conditions. In recent years there has been a proliferation of sleep-focused mobile phone applications, some of which may be suitable for use by shift workers. There is limited evidence however, on whether these applications are sufficient in managing the sleep needs of the early start shift working population (i.e., those whose work schedules begin pre-dawn). This scoping review aims to identify and discuss peer-reviewed literature on mobile sleep applications used by early start shift workers for sleep-self management. Four databases (Scopus, EBSCOhost, CINAHL and PsycInfo) were searched for relevant literature using a pre-determined search string. The initial search using the term *early start shift work* returned no papers, however a broadened search on shift work in general found 945 papers for title and abstract screening, of which 21 were deemed eligible for full text screening. Two of these papers met the inclusion criteria for this review. The results highlight, firstly, the paucity of research on the use of mobile phone applications for sleep self-management amongst early start shift workers, and secondly, the need for further research on the effectiveness of mobile applications for sleep self-management amongst shift workers in general. A working definition of early start shift work that can be used to stimulate research in this understudied population of shift workers is also proposed.

## Introduction

Shift work is an increasingly important employment scheduling practice in a modern society that demands service across all 24 h of the day. Broadly defined, shift work refers to any schedule of work that occurs outside normal daytime working hours. Shift work hours typically consist of either regular nights and evenings or rotating shifts between day and evening hours, although other varieties of shift work also exist. In Australia, approximately 1 in 6 employees works regular shift work hours (1), with equivalent figures reported in European Union member states (2) and the United States of America (3).

Shift work increases risk for multiple health problems if it is not appropriately managed. ‘Shift work disorder', for example, is characterized by symptoms of insomnia and excessive sleepiness and affects approximately a quarter of the shift working population (4). Shift workers are also at increased risk of cardiovascular disease, diabetes, and gastrointestinal disorders (5). Poorer mental health outcomes are also reported in some shift working populations (6). Misalignment of endogenous circadian rhythms with external clock time and reduced sleep time are two possible mediators of the association between shift work and poorer mental health (5). This may be particularly the case for “early start shift workers,” that is, shift workers who begin their work in the pre-dawn period (i.e., before sunrise). From a circadian perspective, this group experience chronic phase misalignment between their endogenous circadian rhythm and typical wake time on workdays (sometimes referred to as *social jetlag* (7). This group are also expected to be sufficiently alert to perform their work duties at a time when their circadian drive for wake is typically at its lowest point in the 24-h circadian cycle. Early start shift workers also experience reduced hours of sleep due to the unusually early bedtime required to achieve a minimum of 7 h sleep at night. Examples of shift workers who typically start work in the pre-dawn period include garbage collectors, bakers, short-haul airline pilots, and some factory workers. In a previous study by our group, we found that racehorse trainers, who also typically start work in the pre-dawn period, reported elevated levels of depression and anxiety (8). Daytime dysfunction due to poor sleep was found to be a potentially important causative factor in their higher levels of psychological distress. Strategies for managing the detrimental effects of poor sleep on health are an essential tool in the shift worker's arsenal of protective behaviors.

The self-management of sleep behavior is an increasingly important strategy for limiting the negative health outcomes associated with compromised sleep. Self-management is especially important for shift workers who commonly experience sleep that is less than optimal in both length and quality (9). With specific reference to early start shift workers, a study of short-haul airline pilots found that those who started their shift before 5 am had significantly reduced sleep hours and higher levels of fatigue than their counterparts who started work later in the day (10). Self-management of health is defined as an “ongoing… process with a focus on self-identified needs or problems that require continual monitoring” (11) p. 1783); it may or may not involve interactions with health care providers. Rapid advances in personalized health-supporting technologies have accelerated the adoption of self-management strategies in both clinical practice and individual efforts to support health, including sleep [e.g., (12)]. Evidence of the useability and effectiveness of these technologies in shift working populations is mixed (13), for example, found that an m-Health app targeting health behavior change in shift workers was rated by users as “slightly to moderately useful” but did not improve sleep quality as intended. Still, increasingly sophisticated, yet user-friendly and affordable technologies mean that technology-mediated self-management of sleep behavior is accessible to a wider demographic than has ever been the case before. Current technologies permit powerful and independent data gathering and use in-built algorithms to facilitate the evaluation of sleep-related phenomena.

Smartphone technologies are particularly suited to the widespread adoption of health self-management strategies. Internationally, the median level of smartphone ownership among advanced economies in 2019 was 76% (14). A more recent consumer survey by Deloitte in Australia reported that 92% of Australians aged 18–75 years owned a smartphone device in 2021 (15). The delivery of sleep health information *via* this medium is therefore accessible to a large proportion of the population. Greater access to smartphones by large sections of the community has led to a proliferation of smartphone apps, including those that encourage self-management of health and well-being. Such growth offers new and more accessible options for health behavior change (16). A large number of smartphone apps specifically designed to support sleep health are available in the major digital marketplaces (e.g., App Store, Google Play) (11) identified just under 2,000 apps that were designed for this purpose. The vast majority consisted of relaxation/meditation sounds or alarm clocks. Apps that offered more than these basic functions varied greatly in quality, content, and functionality. Importantly, none of the apps were designed specifically to support the unusual sleep patterns of shift workers.

The aim of this scoping review was to ascertain the breadth of research available on the use of mobile phone sleep applications for sleep self-management amongst early start shift workers. Preliminary searches yielded no results for the term “early start shift work. As such, the review sought to identify the existing peer-reviewed literature regarding the utilization of mobile sleep applications for the general shift working population. While such studies reflect the results and experiences of shift workers which may at times fall outside of the definition of “early start” shift work, we believe that implications can be drawn from this evidence to support the facilitation of similar studies with a more specific subset of the shift working population—early start shift workers. Further, in light of a lack of consensus on the term “early start shift work,” this scoping review proposes a working definition of this term to aid future research and interventions in broadening understanding of the specific needs of this minority working demographic.

## Methods

### Design

A scoping review methodology was employed and guided by the framework of (17) to ensure a rigorous approach to the investigation.

### Search strategy

Four databases were the subject of a comprehensive search (Scopus, EBSCOhost, CINAHL, and PsycInfo) using the following search string: (“sleep^*^ app^*^” OR “sleep^*^ technolog^*^” OR “sleep^*^ management^*^” OR “Computer and sleep” OR “Computer therapy and sleep” OR “Digital sleep companion” OR “Digital sleep app^*^” OR “Sleep behavio^*^ change and technolog^*^” OR “Sleep monitor^*^ and digital technolog^*^”). These specific databases and search terms were selected due to their likelihood of yielding the broadest range of search results possible.

An initial search was conducted in 2019 by one reviewer (IR) and re-run with the support of a research librarian to ensure accurate results. An additional search was run in 2021 to check for updates to the literature. The initial search in 2019 elicited 1,030 peer reviewed journal papers and the 2020–21 search yielded a further 360 papers. Additional qualifiers using Boolean phrases (AND “shift worker^*^” AND “well-being” OR “well-being” OR “mental health”) were used to seek out literature more specific to the research question, but such phrases yielded no additional results. All papers from the 2009–19 search were added to Endnote, where using the automated and manual deduplication procedure, 430 papers were eliminated. The remaining papers (600) were uploaded to Covidence where 15 were eliminated for being duplicates. The 360 papers from the 2020–21 search were manually screened for duplication and none were eliminated. In total, 945 papers were moved forward to title and abstract screening from which 942 were excluded and 21 progressed to full text screening. After full text screening with consideration for the inclusion and exclusion criteria, a total of 2 papers met the requirements for this scoping review. Twelve of the excluded studies were assessed as being outside of scope (e.g., review paper, mobile phone technology required concomitant use of a wearable device), five of the studies were excluded due to the wrong population being studied (e.g., not shift workers, sleep disorder patients), and a further two studies were excluded because they were not peer-reviewed publications (e.g., expert opinion).

### Article screening

Articles were included in the review if they met the following criteria: 1) were published in English between 2009 and 2021. 2) the study population were shift workers. 3) the study involved the use or analysis of mobile phone sleep applications.

**Figure 1:**
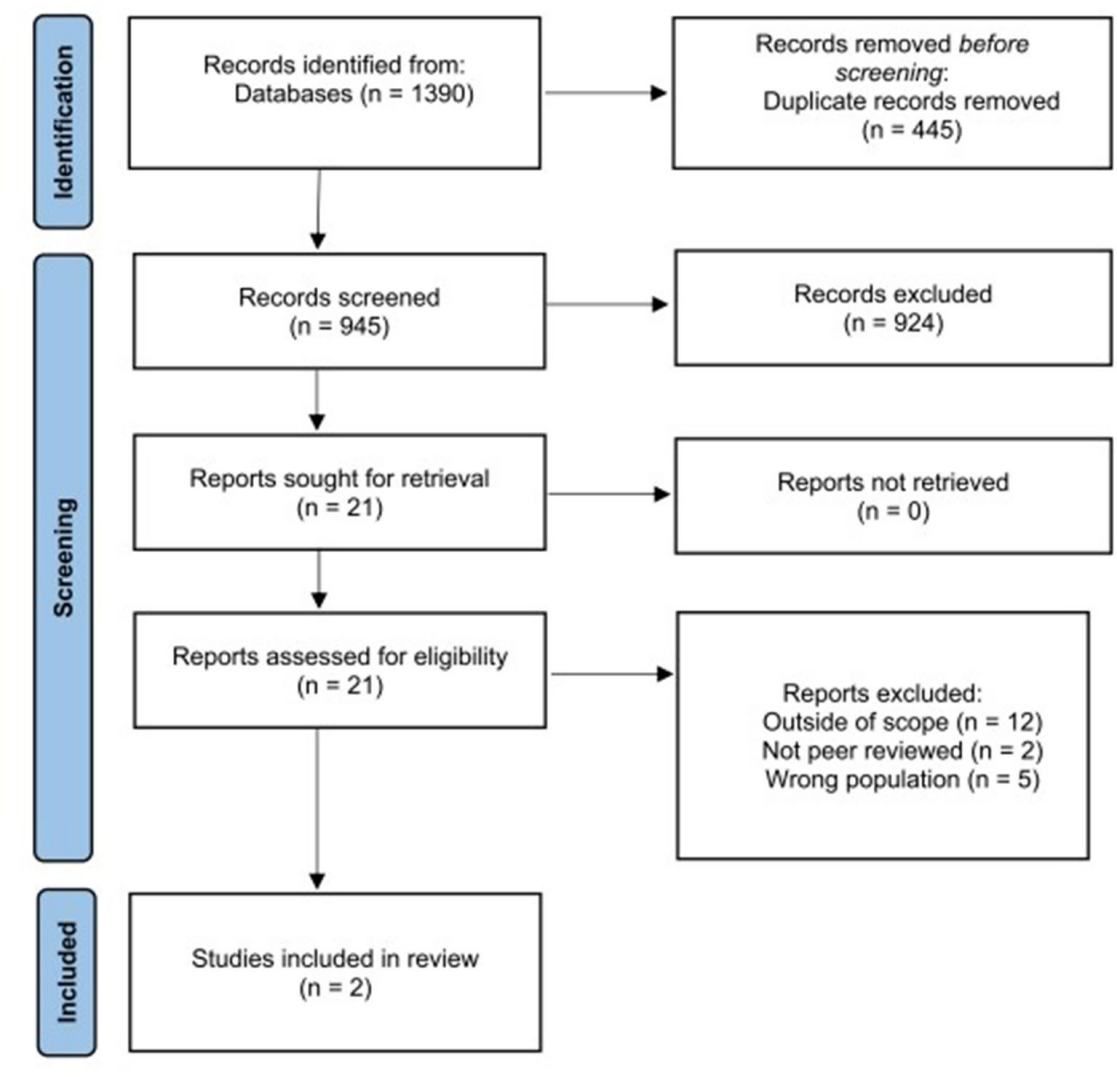
PRISMA diagram showing the flow of selection and exclusion of articles through the different stages of the review.

The following review techniques were employed: 1) only full text peer-reviewed articles were selected. 2) inclusion criteria were limited by date and language. 3) one reviewer (IR) screened and selected the articles, and an additional two secondary reviewers (BB, SH) screened the selection of articles. 3) a secondary reviewer re-ran the search string in all databases to confirm the number of results. 4) a single reviewer (CL) extracted the findings from the studies selected. 5) the PRISMA protocol was followed to guide the development of the scoping review (see Figure 1).

### Data extraction

Using Microsoft Excel, a database was created to manage the articles that made it past the title and abstract screening. Key details from the articles were listed including, title, author(s), year of publication, country of origin, journal of publication, study design, target groups and outcomes. The database was developed and maintained by one reviewer (CL) who extracted the relevant data, and a secondary reviewer (BB) who screened the data and confirmed the inclusion of the selected articles.

## Results

### Characteristics of studies

Two studies met the inclusion criteria for the scoping review. These studies originated from the Republic of Korea (18) and The Netherlands (19), respectively. Both conducted research on the use of mobile sleep applications for shift workers, the first focusing on nurses and the second on airline pilots. The study on nurses employed a qualitative descriptive analysis approach while the study on pilots was a randomized controlled trial. See Table 1 for the extracted data from these studies.

**Table 1:** Overview of studies included in the scoping review.

**Author(s), Year, Country**	**Sample**	**Study design**	**Study aim**	**Key findings**
Joo et al. (18) (Republic of Korea)	Nurses (*N* = 20)	Quantitative descriptive analysis	To study the sleep latency and post-sleep wakefulness of shift working nurses	Time in bed was significantly lower for night shift nurses than day Sleep latency was significantly lower after night shifts than day Sleepiness scores were significantly higher immediately after waking up among night shift nurses
van Drongelen A et al. (19) (The Netherlands)	Pilots (*N* = 502)	Randomized controlled trial	To evaluate the effects of mHealth interventions to improve the health perception of airline pilots	After 6 months the intervention group showed significant improvement on fatigue Offering tailored advice through mHealth is an effective way to support airline staff who work irregular hours and experience circadian disruption

### Study findings

The two included studies investigated the use of mobile phone sleep applications among shift workers, albeit in different ways and in different shift working populations. 18 measured various aspects of sleep (e.g., sleep onset latency, total sleep time, wakefulness during the sleep period) among shift working nurses using the *Sleep Time* app (Azumio Inc.). A sleep diary was used alongside the app for comparison. While the primary outcome of this study was that both sleep onset latency and time in bed were significantly lower on night shift compared to day shift, and sleepiness scores were significantly higher immediately after waking in the night shift group, the outcome most relevant to the current review was the high level of agreement between self-reported and app-assessed sleep parameters (*r* = 0.78 to 0.99). This level of agreement supports the usefulness of objective sleep data collected *via* a mobile phone app as a viable assessment method.

(19) investigated the effectiveness of a health advice intervention on fatigue delivered *via* mobile phone (*MORE Energy* app) to 502 international airline pilots. The health advice was tailored to pilots' flight schedules and personal characteristics (including chronotype) and targeted preventive actions for reducing fatigue, including circadian- and sleep-related behaviors (e.g., optimal timing of exposure to daylight and sleep) and improving fitness and nutrition. The results showed that after 6 months of app use, fatigue was significantly lower among the intervention group compared to a control group of pilots who received generic health advice on the targeted preventive measures. Specific areas of improvement were sleep quality (but not sleep length or sleep latency), healthy snacking behavior, and strenuous physical activity. High levels of app use compliance were required to receive the maximum benefit for reducing fatigue, although moderate improvements were seen even with lower levels of compliance.

## Discussion

This scoping review aimed to investigate previous research on the use of mobile phone applications for sleep self-management amongst early start shift workers. An initial search on this specific employment group failed to locate any studies so we broadened the search to include the general shift working population. Only two studies met study inclusion criteria—one that used an app to investigate sleep among shift working nurses (18) and one that investigated the effectiveness of an app to deliver a fatigue management intervention among international airline pilots (19).

The two included studies make different contributions to the current investigation: 18 showed that sleep data collected *via* the *Sleep Time* mobile phone app were as accurate as self-report sleep diaries for measuring the amount of time spent in bed and the amount of time taken to fall asleep (sleep onset latency). Objective and automated assessment of these sleep characteristics *via* an app may be useful for improving compliance with their measurement, particularly over longer time frames when compliance rates can decline [e.g., (20)]. For shift workers, long-term compliance with the measurement of sleep behavior may be necessary for a comprehensive assessment of how different work schedules and behavior changes affect sleep. The app-based assessment of other sleep characteristics (e.g., total sleep time and the amount of time spent awake after sleep onset) showed less consistency of measurement with the self-report diary. The accuracy of sleep diaries for measuring important sleep characteristics is mixed [e.g., (20, 21)], especially when sleep is compromised [e.g., (22)] or when daytime naps are used [e.g., (23)], both of which may apply in shift working populations. Nevertheless, sleep diaries are often the only viable method available for the self-management of sleep. An automated app that can be used in place of a sleep diary is likely to increase compliance in the long-term and may yield more complex and useful sleep-related information as algorithms continue to improve.

The randomized controlled trial by 19 showed that health advice delivered to shift workers *via* a mobile phone app can successfully initiate and sustain health behavior change. The effectiveness of the app may be attributed to careful tailoring of health advice to the specific characteristics of the user. Generic advice on sleep can be found in many public health forums (e.g., www.sleepfoundation.org) and may be ineffective at producing similar levels of change if it is not tailored to the needs of the target audience [see also, (24)]. Regular engagement with the app also enhanced positive outcomes among users in the van Drongelen, Boot et al. study. To increase long-term compliance, apps should actively encourage engagement through feedback, goal setting, and rewards. These are important considerations in the design of mobile phone apps for facilitating health behavior change [see also, (25)].

The fact that only two studies met inclusion criteria for the review was unexpected, especially after search criteria were broadened to include general shift workers and not just limited to early start shift workers. While the shortage of returned studies may be partially attributed to the search terms used and inclusion/exclusion criteria (see Limitations), this scoping review indicates that there is limited research in this space. Mobile phone apps for sleep are in plentiful supply; however, as reported by (11) few meet minimum expected standards of quality, functionality, and validation for sleep self-management, and even fewer would meet these criteria in a specialist population like shift workers. Application of minimum standards and evidence-based design recommendations [e.g., (11, 16, 25)], are needed. Further, app developers should employ a codesign approach [for instance, see (26)], designing apps *for shift workers with shift workers*, when creating specialist sleep apps. This is particularly the case when designing for specialist workers, such as early start shift workers. This may lead to greater uptake and use of mobile phone apps about sleep self-management and contribute to the body of research in this space.

The complete absence of studies returned from the initial search for early start shift work was also unexpected. As a descriptive term, *early start shift work* appears to be unrecognized in the literature as a way to describe shift work schedules that begin prior to dawn; however, there does not seem to be an alternative description in common use that could apply to this understudied population of shift workers. This group appear to have many unique characteristics that are not shared with other shift workers—rising before dawn when the circadian drive for wake is low, and the associated misalignment of phase between circadian and homeostatic sleep processes, are prominent differences. Another distinctive feature of this group is that early start shift workers have difficulty obtaining sufficient sleep on a regular basis due to the unusually early bedtime required, as is the need to be physically and cognitively alert at times of the day that are biologically prejudicial to such activity. For example, the horse-racing, air traffic control and nursing professions include early start shift workers. In these contexts, errors or lapses in judgement at work may adversely affect the health and well-being of the early start shift worker, and the health and safety of others with whom they work, e.g., people in proximity to horses, airline passengers, and patients. Classifying early start shift workers into the same category as other shift working populations may be disadvantageous to research and policy on the safety and well-being considerations for this minority population of shift workers.

To stimulate research in this understudied population of shift workers we propose a working definition of early start shift work as *a work schedule that starts in the pre-dawn period, that is, before the first light of day*. Light is the most powerful zeitgeber for the endogenous circadian system (27), so appropriately timed exposure to light in the morning is necessary for ensuring a stable 24-h pattern of sleep and wake [see also, (28)]. Tying the definition to morning light schedules instead of 24-h clock time would therefore appear to be an important consideration. From a circadian science perspective at least, clock time is less important than 24-h (circadian) biological time in managing sleep/wake cycles. The proposed definition also has the advantage of being flexible with regards to local time which can vary greatly due to seasonal and latitudinal variations in light/dark cycles. This is especially relevant to shift workers whose work duties are performed mostly in outdoor settings (e.g., horse racing staff and garbage collectors). The proposed working definition may have limited application around the winter solstice in higher northern hemisphere latitudes where dawn occurs later in the morning, meaning that most of the working population would be classified as early start shift workers at these times.

### Limitations

The limitations for this review concern the selected search terms and exclusion criteria. Firstly, inclusion of *smartphone* as a search term may have increased the number of studies returned from the literature search. While we believe most of the relevant literature was captured in our search using terms such as *app, technology*, and *digital*, inclusion of *smartphone* in the search may have been a useful addition. Secondly, studies that employed wearables such as smartwatches, wristbands, and rings in their collection of sleep data were excluded due to the limited availability of these devices in the initial reference period of 2009–2019, and concerns over the application of review findings to the wider population of shift workers. The recent proliferation of wearable technologies in general use, and the enhanced functionality in sleep-related applications such devices afford, means that any update to the current literature review should include research on these devices.

## Conclusion

The findings of this review highlight a paucity of published research on the use of mobile phone applications for sleep self-management amongst early start shift workers. Further research is needed, therefore, on applications appropriate to this subgroup of shift workers whose unique working conditions require specific interventions and support. The appropriate timing and use of light in both the early morning and evening hours is one example of support that is specifically relevant to this group. To stimulate such research, we have proposed a working definition of early start shift work as *a work schedule that starts in the pre-dawn period, that is, before the first light of day*. This definition emphasizes alignment with light/dark cycles and is therefore more consistent with circadian science principles than traditional definitions that emphasize clock time.

We also note that, while a large number of mobile phone applications that target sleep self-management already exist in the digital marketplace, few, if any, are designed specifically for use by shift workers. There is clearly a need, therefore, for a more evidence-based and context-specific approach to the development of mobile phone sleep applications for this group. We recommend that applications are co-designed in partnership with shift workers as the optimal strategy for fulfilling this need.

## Data availability statement

The original contributions presented in the study are included in the article/supplementary material, further inquiries can be directed to the corresponding author.

## Author contributions

BB, HD, and AAM conceived and designed the review. CL collated the study results. BB wrote the manuscript which was reviewed and revised by HD, AAM, and CL. All authors contributed to the article and approved the submitted version.

## Funding

This review was partly funded by an internal grant from Swinburne University of Technology's Social Innovation Research Institute (SIRI).

## Conflict of interest

The authors declare that the research was conducted in the absence of any commercial or financial relationships that could be construed as a potential conflict of interest.

## Publisher's note

All claims expressed in this article are solely those of the authors and do not necessarily represent those of their affiliated organizations, or those of the publisher, the editors and the reviewers. Any product that may be evaluated in this article, or claim that may be made by its manufacturer, is not guaranteed or endorsed by the publisher.
